# Change in the incidence of stillbirth in Japanese Black cattle during the animal movement restrictions and suspended insemination

**DOI:** 10.1371/journal.pone.0304867

**Published:** 2024-06-11

**Authors:** Moe Misaka, Mizuho Uematsu, Go Kitahara, Takeshi Osawa, Yosuke Sasaki

**Affiliations:** 1 Course of Animal and Grassland Sciences, Graduate School of Agriculture, University of Miyazaki, Miyazaki, Japan; 2 Miyazaki Agricultural Mutual Aid Association, Miyazaki, Japan; 3 Department of Veterinary Sciences, Faculty of Agriculture, University of Miyazaki, Miyazaki, Japan; 4 Center for Animal Disease Control, University of Miyazaki, Miyazaki, Japan; 5 School of Agriculture, Meiji University, Kawasaki, Kanagawa, Japan; Bangladesh Agricultural University, BANGLADESH

## Abstract

We aimed to characterize the change in the incidence of stillbirth (IS) in Japanese Black cattle during and after animal movement restrictions and suspended insemination because of a foot-and-mouth disease (FMD) outbreak in Miyazaki Prefecture in 2010. Calving data from 2006 to 2018 were collected from approximately 900 farms. Post-FMD period was divided into three based on the median IS per month (1.80%): period 1 (May 2011 to February 2013), period 2 (March 2013 to August 2015), and period 3 (September 2015 to December 2018). The ISs were similar during the Pre-FMD period and Post-FMD period 1, then substantially decreased during Post-FMD period 2 (*p* < .05), before returning to the value before the FMD outbreak period during Post-FMD period 3. Compared with the Pre-FMD period, Post-FMD period 1 was associated with a higher proportion of calvings by primiparous cows and Post-FMD period 2 was associated with a smaller number of calvings per month (*p* < .05). There were high ISs in primiparous cows during the Pre-FMD period, Post-FMD period 1, and Post-FMD period 3 (*p* < .05), but not during Post-FMD period 2. In summary, after the animal movement restrictions and suspended insemination introduced because of the FMD outbreak, the IS temporarily decreased and consequently returned to the pre-FMD level.

## Introduction

Stillbirth has a significant impact on the productivity of the beef industry [1, 2] and animal welfare [3, 4]. In our previous study, we quantified the incidence of stillbirth (IS) and showed that the risk factors for IS in Japanese Black cattle, which is the most common breed of beef cattle in Japan, are giving birth during winter, primiparity, and short and long gestations [5]. We also found that Japanese Black cows that experience stillbirth show inferior subsequent reproductive performance compared to the other cows [6]. Based on these studies, the producers in the studied region had received advice regarding appropriate calving management by a veterinarian. However, changes of risk factors for IS over time have not been investigated. A longitudinal study examining the temporal pattern of IS can be used to clarify the effect of this point.

In addition to the change of calving management, an occurrence of a foot-and-mouth disease (FMD) outbreak could affect the temporal pattern of IS. FMD is severe global disease [7] and affect economic burden to the livestock farmers. In April 2010, FMD outbreak occurred in Miyazaki prefecture, Japan, and 69,454 cattle were culled and buried. During this outbreak period in Miyazaki prefecture, control measures such as stamping out, movement restrictions, suspended insemination and disinfection of the animals were implemented in the area [8], and all insemination was suspended until July 2010. No study has been investigated on the effect of these control measures on the temporal pattern of IS and the changes of risk factors for IS. In addition, because there have been no outbreaks of FMD in Japan since the resumption of insemination practices after this 3-month period of no practice, this is an excellent model for evaluating the impact of FMD outbreaks on the temporal pattern of IS.

Therefore, in the present study, we aimed to characterize the changes in the IS in Japanese Black cattle during and after the animal movement restrictions and suspended insemination that were introduced during the FMD outbreak in Miyazaki Prefecture in 2010, and to compare the factors for IS before and after the outbreak. We assumed that the animal movement restrictions and suspended insemination affect the frequency of calving and proportion of primiparous cows that would fluctuate IS and consequently risk values in each variable for IS. Results will help to support the design and implementation of prevention and control measures of IS in this breed of cattle.

## Materials and methods

### Study area

The present study was conducted in suburban areas of Miyazaki City in Miyazaki Prefecture, Japan. Miyazaki Prefecture, located on the southeastern coast of Kyushu, is a major beef producing region and has the second largest cattle population in Japan [9, 10]. Miyazaki City is located at 131° 24′ E and 31° 56′ N and has a temperate climate, with warm, humid summers and cold winters. In Miyazaki City, the average temperatures in the summer (July and August) and winter (January and February) were 27.7°C and 8.3°C, respectively, during the study period. The productivity of Japanese Black cattle was the same as in a previous study [9, 11–17]. All the calves were reared in intensive systems and housed in free stalls until weaning, and all of the cows were bred using artificial insemination (AI). Because cattle are not grazed in this region, the cows and calves were fed roughage, such as rice straw, Italian ryegrass, and oat straw, and a dietary concentrate twice daily to meet the Japanese feeding standard requirements for beef cattle. In addition, the calves suckled from the udder until they were separated from their mothers around 120 days after birth.

**Fig 1:**
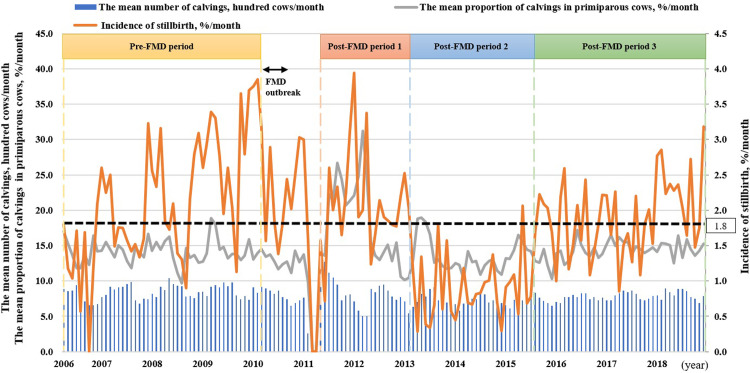
Changes in the monthly incidence of stillbirth, total number of calvings, and proportion of calvings by primiparous cows between April 2006 and December 2018. The Pre-FMD period was between April 2006 and March 2010. Post-FMD periods 1, 2, and 3 were from May 2011 to February 2013, from March 2013 to August 2015, and from September 2015 to December 2018, respectively. Double arrow indicates the period during FMD outbreak (From April to August 2010). Data between September 2010 and April 2011 were excluded because they would have been influenced by the control measures introduced because of FMD.

As a description of FMD outbreak in Miyazaki prefecture in 2010, the index case was diagnosed on a beef-breeding farm on April 20, 2010, and after confirmation of this first case, control measures such as stamping out, movement restrictions, suspended insemination and disinfection were implemented [18]. A total of 292 farms were confirmed positive until the last outbreak on July 4, 2010, during which 69,454 cattle were culled [19]. Most of the farms were recorded in the central area of Miyazaki prefecture (282 of 292), which does not include the study area, but three farms were identified in the study area and 54 animals on these farms and 2,659 animals on 103 farms that had been vaccinated against FMD were euthanized. After confirming the absence of FMD in the affected areas, all movement restrictions were phased out by July 27. Because inseminations were suspended during the outbreak, the number of calves born substantially decreased at the beginning of 2011 (Fig 1).

### Experimental design

Data were collected from all the commercial beef farms in the study area between April 2006 and December 2018. These data were obtained from Miyazaki Agricultural Mutual Aid Association and Miyazaki Prefecture Livestock Association, and obtained the permits from them to use the data. To characterize the changes in the IS in Japanese Black cattle during and after the animal movement restrictions and suspended insemination introduced because of the FMD outbreak and to compare the factors associated with stillbirth before and after the FMD outbreak, we divided the time into Pre-FMD period and Post-FMD period. Consequently, Post-FMD period was further divided into three periods on the basis of the median values of the mean IS per month (1.8%): the period after the FMD outbreak until the month when the incidence was below the median value (Post-FMD period 1); the period after the FMD outbreak, from the month during which the incidence was below the median value to that when it exceeded the median value (Post-FMD period 2); and the period after the FMD outbreak following the month when the incidence exceeded the median value (Post-FMD period 3). During Post-FMD period 2, the IS in this area temporarily decreased, despite no specific control program having been instigated. The Pre-FMD period was between April 2006 and March 2010. Data collected between April 2010 and April 2011 were excluded from the present study because they would have been influenced by the control measures introduced because of FMD. Post-FMD periods 1, 2, and 3 were from May 2011 to February 2013, from March 2013 to August 2015, and from September 2015 to December 2018, respectively (Fig 1).

We collected data from 905 and 899 farms before and after the FMD outbreak, respectively, which included records for 41,184 calvings by 15,512 animals during the Pre-FMD period and 71,098 calvings by 19,975 animals during the Post-FMD period. The data collected during the Pre-FMD period were a subset of a larger dataset that was used in our previous study [5].

The present study was conducted in two parts. In part 1, we used the monthly records to compare parameters such as the IS, total number of births, the number of primiparous cows giving birth, and the proportion of births accounted for by primiparous cows among the four study periods. These monthly records were calculated using individual calving records in each calving month. The number of monthly records in the Pre-FMD period and Post-FMD periods 1, 2, and 3 were 48, 22, 30, and 40 records, respectively. In part 2, we used individual calving records to identify factors associated with stillbirth during each period. There were 41,184 calving records during the Pre-FMD period, 18,035 during Post-FMD period 1, 21,970 during Post-FMD period 2, and 31,093 during Post-FMD period 3.

Because these data were obtained from a regional database, and no experiments were performed on live animals, University Animal Care and Use Committee approval was not required.

### Definitions and categories

Stillbirth was defined as a dead fetus found at calving >240 days after AI [20]. The timing of calving was categorized according to the season: winter (December to February), spring (March to May), summer (June to August), or autumn (September to November). Parity was categorized as 1, 2, 3, 4, 5, 6, 7, 8, 9, or ≥10. The length of gestation was categorized as ≤270 days, 271–280 days, 281–290 days, 291–300 days, or ≥301 days. The proportion of births accounted for by primiparous cows was calculated as the number of primiparous cows that calved, divided by the total number of calvings.

### Statistical analysis

Data were analyzed using SAS software (ver. 9.4; SAS Institute Inc., Cary, NC, USA). For part 1, Pearson correlation analysis was used to analyze the relationships between the IS, the total number of births, the number of primiparous cows giving birth, and proportion of births that was accounted for by primiparous cows. In addition, ANOVA was used to compare the IS, the total number of births, the number of primiparous cows giving birth, and proportion of births that was accounted for by primiparous cows among the Pre-FMD period and Post-FMD periods 1, 2, and 3.

For part 2, a mixed-effects logistic regression model was used to identify factors associated with stillbirth during each period. The dependent variable was whether or not a cow had stillbirth (1 or 0) and the independent variables were calving season, parity, and the length of gestation. We compared to each reference group of these independent variables (reference groups of calving season, parity and gestation length were summer, 5 and 281–290 days, respectively) based on our pervious study [5]. All the possible two-way interactions between significant factors were included, but non-significant interactions (*p* < .05) were removed from the final models. The identity of the farm was included as a random effect. Odds ratios and 95% confidence intervals were calculated for each significant factor.

*p* < .05 was considered to represent statistical significance and 0.05 < *p* < 0.10 was considered to represent a trend.

## Results

For part 1 of the study, the means ± SEMs were 1.81% ± 0.07% for the IS, 802 ± 9.9 total calvings, 118 ± 2.5 calvings by primiparous cows, and a proportion of the number of births accounted for by primiparous cows of 14.7 ± 0.3%. Changes in the monthly IS in each period are shown in Fig 1. Additionally, IS for primiparous and multiparous cows are shown in S1 Fig. Positive correlations were found of the IS with the number of calvings by primiparous cows per month and the proportion of the number of births accounted for by primiparous cows (Table 1; *p* < .05). In addition, there was a trend toward a positive correlation between the IS and total calving number (*p* = .07). Table 2 presents a comparison of these parameters during the four study periods. There was a lower IS during Post-FMD period 2 (*p* < .05) than during the Pre-FMD period, but there were similar incidences during Post-FMD periods 1 and 3. Post-FMD period 2 was associated with the lowest total number of calvings per month (*p* < .05), and Post-FMD period 1 was associated with the highest number of calvings by primiparous cows per month and the highest proportion of births by primiparous cows per month (*p* < .05).

**Table 1:** Correlations of the monthly incidence of stillbirth (IS) with the total number of calvings, the number of calvings by primiparous cows, and the proportion of calvings by primiparous cows.

	Total number of calvings	Number of calvings by primiparous cows	Proportion of calvings by primiparous cows
**IS**	0.15 (*p* = .07)	0.29 (*p* < .05)	0.25 (*p* < .05)
**Total number of calvings**	-	0.60 (*p* < .05)	−0.04 (*p* < .05)
**Number of calvings by primiparous cows**		-	0.76 (*p* < .05)

**Table 2:** Comparison of the monthly incidence of stillbirth (IS), total number of calvings, number of calvings by primiparous cows, and the proportion of calvings by primiparous cows during the Pre-FMD period and Post-FMD periods 1, 2, and 3^a^.

Period^b^	N	IS, %	Total number of calvings	Number of calvings by primiparous cows	Proportion of calvings by primiparous cows, %
**Pre-FMD period**	48	2.15±0.1a	858±14.1a	122±2.9b	14.2±0.2b
**Post-FMD period 1**	22	2.05±0.1a	820±41.9ab	145±10.8a	18.1±1.3a
**Post-FMD period 2**	30	0.90±0.1b	732±13.8c	100±4.0b	13.6±0.4b
**Post-FMD period 3**	40	1.92±0.1a	777±9.6b	112±2.5b	14.4±0.2b

^a^Data are mean ± SEM.

^b^The study periods were defined using the median values of the mean incidence of stillbirth (IS) per month (1.8%): before the FMD outbreak (Pre-FMD period); after the FMD outbreak, until the month when the incidence was below the median (Post-FMD period 1); after the FMD outbreak, from the month when the incidence was below the median to the month when it exceeded the median (Post-FMD period 2); and after the FMD outbreak, from the month when the incidence exceeded the median (Post-FMD period 3).

Values without the same letters (a, b, c) within a column differed significantly (*p* < .05).

For part 2 of the study, Tables 3 and 4 shows the IS, according to calving season, parity, and the length of gestation during each period. During the Pre-FMD period, the IS was associated with calving season, parity, and the length of gestation (*p* < .05). There was a high incidence during winter and spring, in primiparous cattle, and in those with gestational lengths of ≤280 or ≥301 days. Similar results were obtained for Post-FMD period 1 (*p* < .05), but in contrast, during Post-FMD period 2, the IS was associated with calving season and gestational length (*p* < .05), but not with parity. There was a high IS during the autumn and in cows with gestational lengths of ≤280 or ≥301 days. During Post-FMD period 3, the IS was associated with parity and gestational length (*p* < .05), but not with calving season. There was a high IS for cows of parity 1 and 9, and with gestational lengths of ≤280 or ≥301 days. No significant interactions between the included variables were identified in any of the models.

**Table 3:** Multivariable associations of the incidence of stillbirth (IS) and other factors, such as calving season, parity, and gestational length in the period before the FMD outbreak (Pre-FMD period).

	Pre-FMD period		
Variable	N	IS, %	OR (95% CI)
**Calving season** ^**a**^			
**Winter**	9,678	2.92	1.88 (1.54–2.31)
**Spring**	11,127	2.34	1.48 (1.21–1.82)
**Summer**	11,378	1.62	Reference
**Autumn**	9,001	1.76	NS
**Parity**			
**1**	5,854	3.21	1.47 (1.10–1.96)
**2**	5,304	1.96	NS
**3**	4,825	1.97	NS
**4**	4,511	2.08	NS
**5**	4,229	1.92	Reference
**6**	4,091	1.30	NS
**7**	3,742	2.11	NS
**8**	3,201	1.94	NS
**9**	2,375	2.44	NS
**≥10**	3,052	2.33	NS
**Gestational length, days**			
**≤270**	379	53.56	79.0 (62.2–100.4)
**271–280**	1,447	8.64	6.32 (5.06–7.89)
**281–290**	19,883	1.46	Reference
**291–300**	19,023	1.26	NS
**≥301**	452	5.97	4.33 (2.86–6.53)

^a^Calving seasons: Winter (December–February), Spring (March–May), Summer (June–August), and Autumn (September–November).

OR: odds ratio; CI: confidence interval; NS: not significant; Reference indicates the group to which the odds ratio refers

**Table 4:** Multivariable associations of the incidence of stillbirth (IS) and other factors, such as calving season, parity, and gestational length in the period after the FMD outbreak^a^.

	Post-FMD period 1			Post-FMD period 2			Post-FMD period 3		
Variable	N	IS, %	OR (95% CI)	N	IS, %	OR (95% CI)	N	IS, %	OR (95% CI)
**Calving season** ^**b**^									
**Winter**	4,125	2.64	1.60 (1.19–2.16)	3,913	0.64	NS	7,352	2.12	NS
**Spring**	2,911	1.86	NS	6,517	0.98	NS	7,358	1.86	
**Summer**	6,152	1.79	Reference	7,302	0.78	Reference	7,567	1.90	
**Autumn**	4,847	1.96	NS	4,238	1.18	1.51 (1.01–2.25)	8,816	1.78	
**Parity**									
**1**	3,195	3.38	2.19 (1.32–3.64)	2,995	1.60	NS	4,477	3.08	1.70 (1.20–2.39)
**2**	2,673	1.68	NS	3,016	0.80		3,896	2.05	NS
**3**	2,058	1.70	NS	3,370	0.71		3,543	1.27	NS
**4**	1,917	1.46	NS	2,921	1.03		3,505	1.57	NS
**5**	1,696	1.24	Reference	2,276	0.88		3,651	1.51	Reference
**6**	1,567	1.60	NS	1,958	0.56		3,396	1.50	NS
**7**	1,365	2.05	NS	1,671	1.02		2,769	2.02	NS
**8**	1,174	1.70	NS	1,371	0.36		2,075	1.54	NS
**9**	900	1.44	NS	1,017	0.88		1,473	2.31	1.59 (1.00–2.53)
**≥10**	1,490	3.02	1.83 (1.03–3.24)	1,375	0.58		2,308	2.08	NS
**Gestational length, days**									
**≤270**	129	62.02	104.5 (68.6–159.2)	94	30.85	53.3 (31.5–89.9)	223	54.71	76.2 (55.9–103.8)
**271–280**	399	13.28	9.23 (6.49–13.14)	337	8.31	10.1 (6.3–16.0)	590	14.92	10.8 (8.2–14.2)
**281–290**	7,169	1.65	Reference	8,080	0.85	Reference	11,941	1.62	Reference
**291–300**	10,064	1.05	0.64 (0.49–0.84)	13,074	0.47	0.57 (0.40–0.80)	17,758	1.01	0.63 (0.51–0.78)
**≥301**	274	4.01	2.56 (1.35–4.88)	385	2.08	2.61 (1.23–5.54)	581	2.07	NS

^a^The periods were defined using the median values of the mean incidence of stillbirth (IS) per month (1.8%): after the FMD outbreak, until the month when the incidence was below the median (Post-FMD period 1); after the FMD outbreak, from the month when the incidence was below the median to the month when it exceeded the median (Post-FMD period 2); and after the FMD outbreak, from the month when the incidence exceeded the median (Post-FMD period 3).

^b^Calving seasons: Winter (December–February), Spring (March–May), Summer (June–August), and Autumn (September–November).

OR: odds ratio; CI: confidence interval; NS: not significant; Reference indicates the group to which the odds ratio refers

## Discussion

The present study is the first to characterize the IS during Pre-FMD and Post-FMD periods, and we have identified associated risk factors during each period. However, it is noteworthy that the present study was an observational study analyzed the data collecting from commercial farm condition, and had several limitations that should be noted when interpreting the results. First, results obtained in part 1 and 2 should not be interpreted as causality, but as association. Second, other relevant parameters, such as nutritional condition, the effect of sire, cow age, and management before and after the FMD outbreak could not be evaluated. Although the environment and management before and after FMD outbreak have not dramatically changed, further research, including studies in other areas, should be conducted to determine more about the association between the duration of the animal movement restrictions (duration of suspended insemination) and the IS.

Results of temporal pattern of IS showed that after the animal movement restrictions and suspended insemination introduced because of the FMD outbreak, the IS temporarily decreased in which a low incidence lasted for 2 years and it returned to the pre-FMD level. The IS during Post-FMD period 2 (0.9%) was lower than that published previously [5, 21]. Additionally, the IS in winter during Post-FMD period 2 was lower than those in any seasons in the other period. There are several possible reasons for low IS. First, results of part 1 showed the lower mean number and proportion of calvings by primiparous cows per month during this period. Primiparity has previously been identified as a risk factor for stillbirth in Japanese Black cattle [5]. Mee [22] reported that the principal types of dystocia differ between primiparous and multiparous cows, with fetal-pelvic disproportion, caused by a large calf relative to maternal pelvic size. Second, results of part 1 also showed the lower mean number of calvings per month during this period. The preventive measures instituted during the FMD outbreak, such as culling and burial [8], and the cessation of artificial insemination between April 2010 and August 2010, might have resulted in extra space being available in the cattle barn. This might have facilitated the provision of additional care for primiparous cows.

In contrast to Post-FMD period 2, the IS during Post-FMD period 1 was similar to that during the Pre-FMD period. Possible reasons for this result would be the results of part 1 showing that the number of calvings by primiparous cows and the proportion of calvings by primiparous cows during Post-FMD period 1 were higher than those during the Pre-FMD period. The first-service conception rate at first calving was higher than at multiparous [9, 11]. However, primiparity is a risk factor for stillbirth, and there was a large number of primiparous cows during Post-FMD period 1. During Post-FMD period 3, the incidence returned to the Pre-FMD level, because the total number of calvings and the proportion of primiparous cows calving had returned to their Pre-FMD values. In Japan, Japanese Black herd size and the number of cows per farm have been increasing [23], and a recent study in Miyazaki revealed that most farmers intend to increase the number of Japanese Black cattle after the FMD outbreak [24]. Thus, these findings indicate that the distribution of parity was temporarily changed because of the FMD outbreak, but this change gradually disappeared.

Primiparous cows had a higher IS than multiparous cows during the Pre-FMD period and Post-FMD periods 1 and 3, but not during Post-FMD period 2. Possible reasons for this result would be the results of part 1 showing that fewer number of calvings during the Post-FMD period 2. Infrequent calving enables producers to provide more care for individual cows [13]. The use of ICT devices can reduce the incidence of accidents at calving from 2.2% to 0.3% [25]. In addition, a calving predictive device is an effective means of predicting the time of calving and reducing the risk of fetal death [26, 27]. Thus, we recommend that producers check whether a calving accident has occurred using an ICT device before a visit by a veterinarian, regardless of the mean number of calvings per month on the farm.

The IS was higher in cows that had been pregnant for ≤280 days or ≥301 days than in those that calved between 281 and 290 days of pregnancy during all the study periods. The present findings indicate that the animal movement restrictions and suspended insemination introduced because of the FMD outbreak had almost no influence on the effects of long and short gestations on stillbirth. In addition, both long and short gestational lengths, which can be the result of factors such as growth retardation of the fetus [20] or an excessively large fetus [28], were risk factors for stillbirth.

The IS during winter and spring was higher than that during summer during the Pre-FMD period and Post-FMD period 1, but calving season was not associated with IS during Post-FMD periods 2 or 3, probably because the cows received less perinatal care during winter. In our previous study, we showed that calving during winter is a risk factor for stillbirth in Japanese Black cattle. In fact, the producers who participated in the present study had received advice regarding appropriate calving management by a veterinarian on the basis of the recommendations by Uematsu *et al*. [5]. Although the IS during Post-FMD period 3 was high year-round, we recommend that producers should maintain a high level of care around calving during winter and spring to reduce the risk of stillbirth.

In conclusion, we have shown that after the animal movement restrictions and suspended insemination introduced because of the FMD outbreak in Miyazaki Prefecture in 2010, the IS in Japanese Black cattle temporarily decreased in which a low incidence lasted for 2 years between 2013 and 2015, and it returned to the Pre-FMD level. The period with low IS also showed the lowest number of monthly calving, and disappeared high risk of IS at winter and primiparous calvings. High risks of IS at long and short gestations were found in both Pre-FMD and Post-FMD periods.

## Supporting information

S1 Fig Changes in the monthly incidence of stillbirth for primiparous cows and multiparous cows, total number of calvings, and proportion of calvings by primiparous cows between April 2006 and December 2018. The Pre-FMD period was between April 2006 and March 2010. Post-FMD periods 1, 2, and 3 were from May 2011 to February 2013, from March 2013 to August 2015, and from September 2015 to December 2018, respectively. Double arrow indicates the period during FMD outbreak (From April to August 2010). Data between September 2010 and April 2011 were excluded because they would have been influenced by the control measures introduced because of FMD.
(TIF)
