# Supplementary figures and images for: Change in the incidence of stillbirth in Japanese Black cattle during the animal movement restrictions and suspended insemination
Source: PLoS One. 2024 Jun 11;19(6):e0304867. doi: 10.1371/journal.pone.0304867 (PMC11166273; doi:10.1371/journal.pone.0304867)

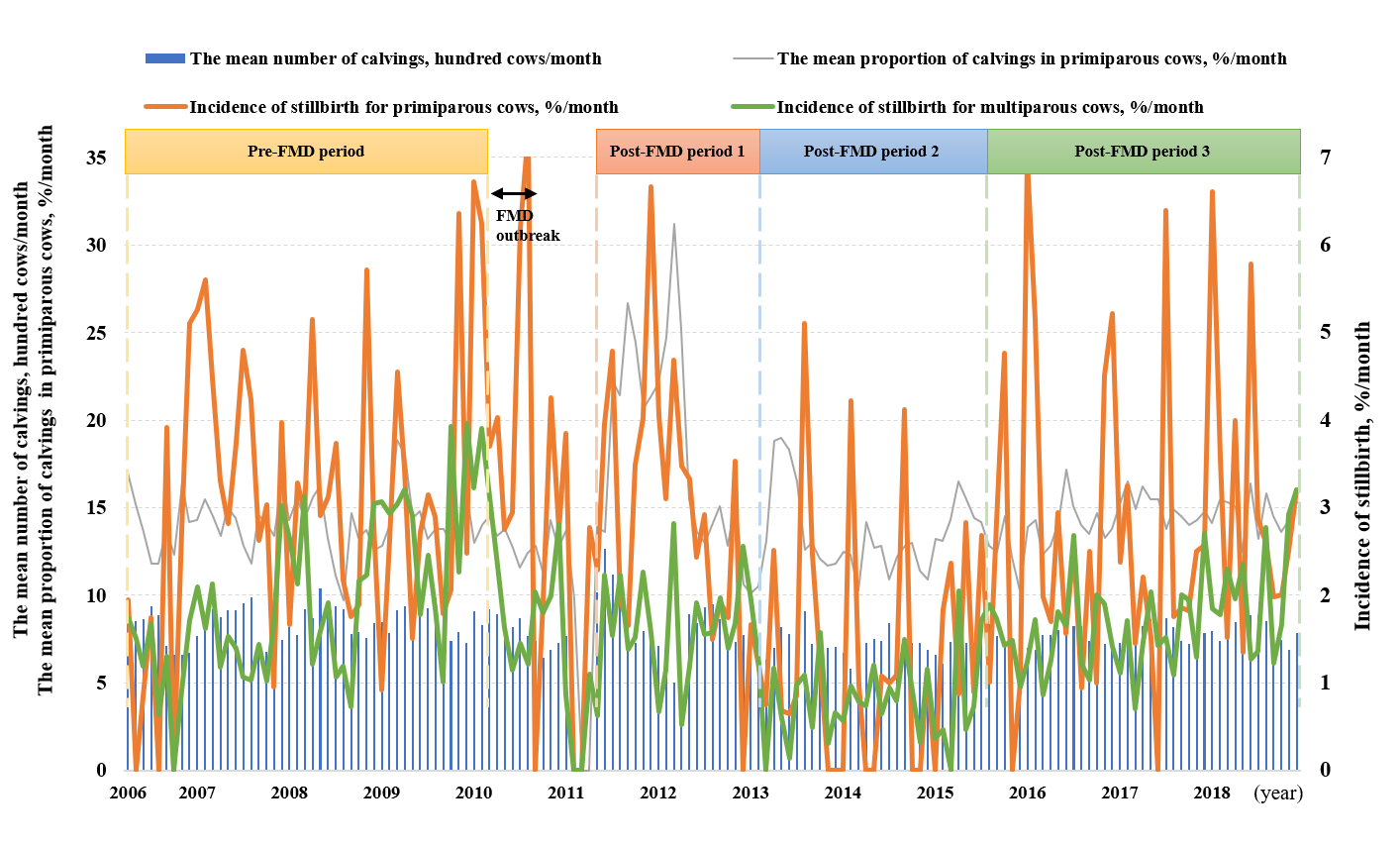

Supplement: S1 Fig — The Pre-FMD period was between April 2006 and March 2010. Post-FMD periods 1, 2, and 3 were from May 2011 to February 2013, from March 2013 to August 2015, and from September 2015 to December 2018, respectively. Double arrow indicates the period during FMD outbreak (From April to August 2010). Data between September 2010 and April 2011 were excluded because they would have been influenced by the control measures introduced because of FMD. (TIF) [file pone.0304867.s001.tif]
